# The Effect of Body Mass Index on Outcome Following Ambulatory High Ligation and Stripping for Lower Varicose Veins: A Prospective Cohort Study

**DOI:** 10.3389/fsurg.2022.801729

**Published:** 2022-04-04

**Authors:** Chu Wen Chen, Yu T. Cai, Jia R. Wang, Zhou P. Wu, Yang Liu, Bing Huang, Yi Yang, Ding Yuan, Yu K. Ma, Ji C. Zhao

**Affiliations:** ^1^ Department of vascular surgery, West China Hospital, Sichuan University, Chengdu, China; ^2^ Department of ambulatory center, West China Hospital, Sichuan University, Chengdu, China

**Keywords:** varicose vein, high ligation and stripping, body mass index, ambulatory care center, great saphenous vein, prospective cohort study

## Abstract

**Objectives:**

The effects of body mass index (BMI) on the outcome of high ligation and stripping (HLS) in an ambulatory center remain unclear. This study aims to investigate the outcomes of HLS in an ambulatory center based on BMI in the Chinese population.

**Design:**

This was a prospective cohort study with mid-term follow-up.

**Materials and Methods:**

170 eligible patients were included in the study and the data of Clinical, Etiology, Anatomy, and Pathophysiology (CEAP) classification, Venous Clinical Severity Score (VCSS), Visual Analogue Score (VAS), Aberdeen Varicose Veins Questionnaire (AVVQ), Quality of Recovery (QoR-15), and postoperative complications at predetermined time points were collected.

**Results:**

A total of 170 patients (236 limbs) with a mean age of 53.87 ± 9.96 years (range, 24–80 years) and a mean BMI of 23.86 ± 2.96 kg/m^2^ were included. Of the group, 50.6% were women, and 66 patients received bilateral procedures. Through curve fitting, a BMI less than 28 and a BMI of 28 or higher were found to have a negative [−0.1 (−0.3, 0.1) 0.296] and positive [0.7 (0.2, 1.2) 0.006] relationship trend, respectively, with the improvement of VCSS at 6 weeks after surgery. Through smooth curve fitting, BMI was shown to have a negative relationship trend on the improvement of VCSS at 6 months after surgery. After multivariable risk adjustment for potential confounding factors, BMI was not found to be associated with the improvement of VCSS and AVVQ at 6 weeks after surgery, as well as the improvement of AVVQ at 6 months after surgery (all *p*-values >0.05). Six months after surgery, BMI was shown to have a negative relationship trend on the improvement of VCSS, and obese patients showed lower VCSS improvement than patients of normal BMI [−1.3 (−1.9, −0.7) <0.0001]. Six weeks after surgery, postoperative complications such as paresthesia were found to be significantly higher in the obese group than in the non-obese group (*p* < 0.05). At 6 months after surgery, the obese group showed significantly higher complications of the legs compared with the normal BMI group (*p* < 0.05).

**Conclusions:**

Our results showed that obesity is a risk factor for prognosis and postoperative complications following ambulatory HLS.

## Introduction

Varicose vein disease is a common condition presenting to physicians globally, and approximately 25 to 40% of the world’s population is affected by it (1–4). The Society for Vascular Surgery/American Venous Forum evidence-based guidelines recommend endovenous laser ablation (EVLA)/radiofrequency ablation (RFA) as the primary treatment (1 B) (5). However, due to factors such as its cost, partial coverage by medical insurance, ineffectiveness for severely distorted veins, and the need for expensive equipment, EVLA/RFA has not been widely used, especially in developing countries. High ligation and stripping (HLS), on the other hand, is a classic treatment that has numerous advantages and is widely used, especially in developing countries (6). Numerous studies have reported that HLS performed in ambulatory centers is just as safe and feasible as HLS performed in hospitals (7–9). In particular, HLS performed in ambulatory centers has several advantages, including lower costs, quicker recovery, improved patient satisfaction and comfort, and much shorter hospitalization time (10–12).

Some studies have reported that overweight patients undergoing HLS have a higher rate of lymphatic complications and wound infections (13–14), and should be monitored closely in the perioperative period. Another study also demonstrated that moderate and severe obesity were associated with an increased incidence of postoperative complications and unplanned admissions in major ambulatory centers (13–15). Despite some evidence suggesting that increased weight can promote the incidence of lower limb varicose veins, there are few studies focusing on the effects of weight on patients after HLS in ambulatory centers. According to the World Health Organization criteria (16), a body mass index (BMI) of 25.0 kg/m^2^ or higher is defined as overweight; however, this cut-off point may not be appropriate for Asian populations. According to the Chinese national standard (17), a BMI of 24.0 kg/m^2^ or higher is defined as overweight and a BMI of 28.0 kg/m^2^ or higher is defined as obesity. Thus, the study aimed to prospectively investigate the early and mid-term outcomes based on the BMI in a Chinese tertiary-care institution, and determine the correlation between BMI and the prognosis of HLS performed in an ambulatory center. Our hypothesis was that overweight and obese patients had worse clinical outcomes after HLS in an ambulatory center.

## Materials and Methods

A prospective cohort study was performed with 170 eligible patients who underwent their first HLS for varicose veins in an ambulatory center from November 2016 to October 2017 in our hospital. Our research protocol was approved by the West China Hospital Ethics Committee and was in accord with the ethical guidelines of the Declaration of Helsinki.

We included patients with C2-4 classes of venous disease (advanced CEAP classification), who were less than 85 years of age, diagnosed with varicose veins by ultrasound, and willing to sign an informed consent. Patients are routinely examined in the standing position and reverse flow of greater than 0.5 m/s following a compression of the calf or valsalva manoeuvre is considered pathological reflux. Patients with any of the following criteria were excluded from this study: 1. Great saphenous varicose vein recurrence; 2. secondary varicose veins (post-thrombotic syndrome); 3. small saphenous varicose veins; 4. acute deep vein thrombosis; 5. superficial phlebitis; 6. Incompetent perforating vein; 7. varicose veins from trauma; and 8. severe comorbidity.

We collected data on the Venous Clinical Severity Score (VCSS) (18), the Aberdeen Varicose Veins Questionnaire (AVVQ) (19), Visual Analogue Score (VAS) (20), Quality of Recovery-15 (QoR-15, QoR15 consists of part QOR15-part A and part QOR15-part B) (21), and the Clinical, Etiology, Anatomy, and Pathophysiology (CEAP) classification (22) before surgery and during postoperative follow-up. The improvement in VCSS and AVVQ were our primary endpoints. The incidence of systemic and leg-specific complications in both groups were our secondary endpoints and were recorded at 6 weeks and 6 months post-procedure. All outcomes were assessed by the same certified specialists in the study team. Our research protocol was approved by the local committee and was in accord with the ethical guidelines of the Declaration of Helsinki. All patients provided written informed consent. Meanwhile, our research was registered in the Chinese Clinical Trial Registry (ChiCTR-ORC-17011614).

### Surgical Procedure

The standard HLS procedure was performed under general anesthesia (without tumescent anesthesia) by the same group of surgeons (chief physicians). The micro-incisions (2–3 cm) were made parallel to the dermatoglyphics with a proximal oblique incision in the groin and a distal transverse incision in the medial malleolus, when deemed necessary (i.e., when the great saphenous vein (GSV) was dilated with severe reflux all the way distally). The trunk and tributaries were systematically and thoroughly treated by ligation. A standard stripper was inserted in the GSV and the vein was stripped from the top down to just below the knee. Another small stripper was inserted in the distal transverse incision of the GSV in the medial malleolus and the vein was stripped from the bottom upward to near the knee, when deemed necessary (i.e., when the GSV was dilated with severe reflux all the way distally). When indicated, phlebectomy of the marked varicose branches and ligation of the grossly incompetent perforators were performed simultaneously. The groin and distal incisions were closed by an intradermal continuous suture (Monocryl@ 5/0, Ethicon, Johnson & Johnson, Neuchâtel, Switzerland). At the end of the surgical procedure, the leg was wrapped in sterile absorbent gauze and covered with a single-layer elastic bandage. After 48 h, the patient could remove the bandage and was allowed to walk as soon as possible. A Class II (30 mmHg) below-knee elastic stocking was used for 3 months during the daytime only. No thermal ablation or sclerotherapy was performed during the surgical procedure. Any additional procedures during the follow-up, such as surgery, thermal ablation, or sclerotherapy, were considered a failure, and these patients were not included in the study. We do not routinely use heparin after ambulatory HLS; only patients with a high risk of thrombosis (Caprini score ≥3) receive heparin (subcutaneous injection of low-molecular-weight heparin 4,000 IU/day) to prevent thrombosis (23). We routinely used early ambulation and compression therapy to prevent thrombosis.

### Follow-up

All the patients were seen twice: at 6 weeks and 6 months after the procedure. Postoperative complications (systemic and leg-specific complications), CEAP class, VCSS score, AVVQ score, VAS score, and QOR-15 score were evaluated and recorded. Follow-up methods included outpatient visits, interviews by telephone and email, and online forums.

### Statistical Analysis

We first described the demographics and general characteristics of the study participants. Then, univariate analysis and stratified analysis were performed to detect the risk variables associated with the BMI group and the improvement of VCSS, as well as AVVQ, at 6 weeks and 6 months after surgery. We further applied a two-piecewise linear regression model to examine the threshold effect of the BMI on the improvement of VCSS and AVVQ at 6 weeks and 6 months after surgery using a smoothing function. The threshold level (i.e., turning point) was determined using trial and error, including a point that gave the maximum model likelihood. We also conducted a log-likelihood ratio test comparing the one-line regression model with a two-piecewise linear model. The Pearson test was used for analysis of the correlation between BMI and the improvement of VCSS and AVVQ. Finally, multivariate regression analysis was performed between the BMI groups. All data were double entered and then exported to tab-delimited text files. The qualitative variables were presented as numbers and percentages, and the quantitative variables were expressed as mean and standard deviation (SD). A *p*-value of <0.05 was considered statistically significant. All the analyses were performed with R (http://www.R-project.org) and EmpowerStats software (www.empowerstats.cm, X&Y solutions, Boston, MA, USA).

## Results

A total of 204 ambulatory HLS procedures were performed from January 2016 to December 2017 and 170 eligible patients were enrolled in this study. We excluded 21 patients for additional procedures (e.g., foam sclerotherapy/endovascular radiofrequency ablation) and 13 patients who were lost to follow-up. The mean age of the patients was 53.87 ± 9.96 years (range, 24–80 years), and the mean BMI was 23.86 ± 2.96 kg/m^2^. Of the patient group, 50.6% were women, and 66 patients received bilateral procedures. The proportion of men in the high BMI group was significantly higher than that of women (*p* = 0.004). The overweight group and obese group had higher VAS (*p* = 0.023) and AVVQ scores than the normal group (*p* = 0.047). Apart from these three factors, there were no significant differences in the basic characteristics of the BMI groups (Table 1).

**Table 1:** Demographics and general characteristics of study participants (*n* = 170).

BMI Group	normal (BMI < 24)	Overweight (24 ≤ BMI < 28)	Obese (BMI ≥ 28)	*p*-value
	*N* = 96	*N* = 59	*N* = 15	
	$\bar{X}$ ± SD/ NO. (%)	$\bar{X}$ ± SD/ NO. (%)	$\bar{X}$ ± SD/NO. (%)	
BMI (kg/m^2^)	21.9 ± 1.5	25.5 ± 1.0	30.1 ± 2.5	<0.001*
Age (years)	52.9 ± 11.2	54.6 ± 8.0	57.3 ± 7.5	0.213
Gender (%)				0.004*
Female	59 (61.5)	23 (39.0)	4 (26.7)	
Male	37 (38.5)	36 (61.0)	11 (73.3)	
Limbs (%)				0.571
Unilateral	57(59.4)	39 (66.1)	8 (53.3)	
Bilateral	39 (40.6)	20 (33.9)	7 (46.7)	
Diabetes (%)				0.206
Non-diabetes	92 (95.8)	59 (100.0)	15 (100.0)	
Diabetes	4 (4.2)	0 (0.0)	0 (0.0)	
Hypertension (%)				0.439
Non-hypertension	84 (87.5)	54 (91.5)	12 (80.0)	
Hypertension	12 (12.5)	5 (8.5)	3 (20.0)	
Preoperative CEAP classification (%)				0.44
2	33 (34.4)	22 (37.3)	3 (20.0)	
3	17 (17.7)	6 (10.2)	4 (26.7)	
4	46 (47.9)	31 (52.5)	8 (53.3)	
Preoperative VAS (%)				0.023*
0	12 (12.5)	3 (5.1)	2 (13.3)	
1	50 (52.1)	22 (37.3)	5 (33.3)	
2	24 (25.0)	28 (47.5)	5 (33.3)	
3	7 (7.3)	2 (3.4)	0 (0.0)	
4	3 (3.1)	3 (5.1)	3 (20.0)	
5	0 (0.0)	1 (1.7)	0 (0.0)	
Preoperative VCSS (%)				0.227
1	1 (1.0)	0 (0.0)	0 (0.0)	
2	1 (1.0)	1 (1.7)	0 (0.0)	
3	5 (5.2)	3 (5.1)	0 (0.0)	
4	12 (12.5)	10 (16.9)	2 (13.3)	
5	23 (24.0)	11 (18.6)	3 (20.0)	
6	13 (13.5)	12 (20.3)	3 (20.0)	
7	17 (17.7)	12 (20.3)	1 (6.7)	
8	8 (8.3)	2 (3.4)	0 (0.0)	
9	9 (9.4)	4 (6.8)	1 (6.7)	
10	1 (1.0)	2 (3.4)	2 (13.3)	
11	0 (0.0)	1 (1.7)	1 (6.7)	
12	4 (4.2)	0 (0.0)	2 (13.3)	
13	0 (0.0)	0 (0.0)	1 (6.7)	
14	1 (1.0)	0 (0.0)	0 (0.0)	
15	1 (1.0)	0 (0.0)	0 (0.0)	
16	0 (0.0)	1 (1.7)	0 (0.0)	
Preoperative AVVQ	13.2 ± 7.1	14.5 ± 7.4	18.3 ± 10.5	0.047*
Operative time	56.0 ± 18.1	59.6 ± 20.5	66.7 ± 36.6	0.158
Preoperative QOR15A	79.3 ± 16.1	80.4 ± 14.1	82.2 ± 12.2	0.76
Preoperative QOR15B	42.5 ± 6.7	43.6 ± 7.3	42.7 ± 6.0	0.641

*QoR15A, Quality of Recovery part A; QoR15B, Quality of Recovery part B;* $\bar{X}\;\pm \;SD$*, mean and standard deviation.*

*Statistically significant.

We performed univariate analysis to detect the effects of BMI risk factors on the improvement of AVVQ and VCSS at 6 weeks and 6 months after surgery. The univariate regression analysis showed that the improvement of AVVQ at 6 weeks and 6 months after surgery was significantly correlated with bilateral HLS and CEAP classification 4 (all *p* < 0.05). Preoperative QoR15B had a negative relationship trend on the improvement of VCSS at 6 weeks and 6 months after surgery, especially in the group with high preoperative QoR15B scores (Table 2). A positive relationship trend was found between CEAP classification and the improvement of VCSS at 6 weeks and 6 months after surgery (Table 2). In the overweight group, we found that BMI had a positive relationship trend on the improvement of AVVQ at 6 months after surgery [4.8 (0.5, 9.0) 0.0298].

**Table 2:** Effects of risk factors of BMI on the improvement of AVVQ and VCSS at six weeks and six months after surgery by univariate analysis.

**Exposure**	**Statistics (*n* = 170)**	**VCSS improvement (6W) β (95% CI) *p* value**	**VCSS improvement (6M) β (95% CI) *p* value**	**AVVQ improvement (6W) β (95% CI) *p* value**	**AVVQ improvement (6M) β (95% CI) *p* value**
BMI Group					
normal (<24)	96 (56.5%)	0	0	0	0
overweight (≤24, >28)	59 (34.7%)	0.2 (−1.1, 0.8) 0.758	−0.6 (−1.5, 0.2) 0.147	−0.1 (−3.0, 2.7) 0.928	0.2 (−1.5, 0.96) 0.980
obese (≥28)	15 (8.8%)	0.8 (−0.8, 2.4) 0.334	−0.1 (−1.5, 1.4) 0.925	3.0 (−1.8, 7.9) 0.218	4.8 (0.5, 9.0) 0.0298*
Age /years	53.9 ± 10.0	−0.09 (−0.77, 0.021) 0.26	0.0002 (−0.04, 0.042) 0.69	−0.102 (−0.24, 0.06) 0.233	−0.12 (0.22, 0.039) 0.168
Gender					
Female	86 (50.6%)	0	0	0	0
Male	84 (49.4%)	−0.4 (−1.3, 0.5) 0.432	−0.3 (−1.1, 0.5) 0.522	−0.3 (−3.0, 2.4) 0.817	−0.8 (−3.2, 1.6) 0.51
Limbs					
Unilateral	104 (61.2%)	0	0	0	0
Bilateral	66 (38.8%)	0.1 (−0.8, 1.0) 0.809	0.4 (−0.5, 1.2) 0.370	4.6 (1.9, 7.2) 0.0009*	4.6 (2.2, 6.9) 0.0002*
Diabetes					
Non-diabetes	166 (97.6%)	0	0	0	0
Diabetes	4 (2.4%)	−0.4 (−3.3, 2.6) 0.808	1.1 (−1.6, 3.7) 0.435	−5.8 (−14.6, 2.9) 0.193	−2.3 (−10.1, 5.6) 0.57
Hypertension					
Non-hypertension	150 (88.2%)	0	0	0	0
Hypertension	20 (11.8%)	0.2 (−1.2, 1.6) 0.766	0.5 (−0.8, 1.8) 0.435	0.9 (−3.2, 5.1) 0.655	0.5 (−3.2, 4.2) 0.798
Preoperative CEAP classification					
2	58 (34.1%)	0	0	0	0
3	27 (15.9%)	1.4 (0.1, 2.6) 0.0370*	1.8 (0.7, 2.9) 0.0021*	2.4 (−1.6, 6.4) 0.248	2.8 (−0.8, 6.4) 0.129
4	85 (50.0%)	2.5 (1.6, 3.5) <0.0001*	2.7 (1.9, 3.5) <0.0001*	3.6 (0.7, 6.5) 0.016*	3.1 (0.5, 5.7) 0.020*
Preoperative VAS					
0	17 (10.0%)	0	0	0	0
1	77 (45.3%)	−0.92 (−2.1, 0.98) 0.478	0.6 (−0.8, 2.0) 0.419	1.1 (−3.5, 5.8) 0.630	−0.2 (−4.3, 3.9) 0.927
2	57 (33.5%)	−0.2 (−1.9, 1.4) 0.774	0.3 (−1.2, 1.7) 0.731	−0.1 (−4.9, 4.7) 0.956	0.0 (−4.3, 4.3) 0.999
3	9 (5.3%)	1.9 (−0.6, 4.3) 0.133	1.7 (−0.5, 3.8) 0.137	−1.3 (−8.5, 5.8) 0.716	−0.3 (−6.6, 6.1) 0.932
4	9 (5.3%)	0.7 (−1.7, 3.2) 0.546	1.1 (−1.1, 3.3) 0.323	6.3 (−0.9, 13.4) 0.089	6.5 (−0.4, 12.9) 0.068
5	1 (0.6%)	−3.6 (−9.6, 2.4) 0.244	−4.2 (−9.7, 1.2) 0.127	4.1 (−13.8, 22.0) 0.654	6.2 (−9.7, 22.1) 0.445
Preoperative QOR15A	80.0 ± 15.1	0.089 (0.014, 0.049) 0.271	0.04 (−0.021, 0.034) 0.62	0.11 (−0.032, 0.16) 0.19	0.11 (−0.03, 0.14) 0.189
Preoperative QOR15B	42.9 ± 6.8	−0.141(−0.14, −0.042) 0.039*	−0.16 (−0.13, −0.004) 0.038*	−0.1 (−0.3, 0.1) 0.184	−0.4 (−3.4, −1.7.0) 0.725
Preoperative QOR15B Tertile					
Low	51 (30.0%)	0	0	0	0
Middle	62 (36.5%)	−0.5 (−1.6, 0.6) 0.391	−1.0 (−2.0, 0.0) 0.048*	−1.8 (−5.1, 1.5) 0.277	−1.8 (−4.7, 1.1) 0.227
High	57 (33.5%)	−1.2 (−2.3, 0.0) 0.045*	−1.2 (−2.2, −0.2) 0.022*	−1.5 (−4.9, 1.8) 0.369	−2.4 (−5.4, 0.6) 0.119

QoR15A: Quality of Recovery part A; QoR15B: Quality of Recovery part B; VCSS improvement (6W): the improvement of VCSS at six weeks after surgery; AVVQ improvement (6W): the improvement of AVVQ at six weeks after surgery; VCSS improvement (6M): the improvement of VCSS at six months after surgery; AVVQ improvement (6M): the improvement of AVVQ at six months after surgery. *Statistically significant. β: Beta coefficient.

**Figure 1:**
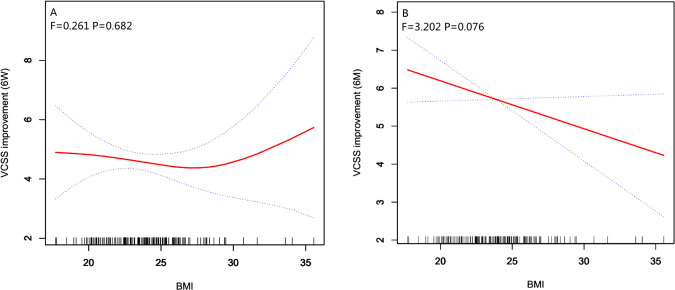
Smooth curve fitting for the relationship between BMI and the improvement of VCSS at 6 weeks and 6 months after surgery *. *: Adjust I adjust for: Gender; Years; Limbs; Preoperative CEAP; Preoperative AVVQ; Preoperative VAS; Preoperative QoR15-A; Preoperative QoR15-B.

We further applied a two-piecewise linear regression model to examine the threshold effect of BMI on the improvement of VCSS at 6 weeks and 6 months after surgery using a smoothing function (Table 3 and Figure 1). Through curve fitting, a negative relationship trend was found between BMI less than 28 and the improvement of VCSS at 6 weeks after surgery [−0.1 (−0.3, 0.1) 0.296]. Conversely, a positive relationship trend was found between BMI of 28 or higher and the improvement of VCSS at 6 weeks [0.7 (0.2, 1.2) 0.006], (the P value of log-likelihood ratio (PLLR): *p* = 0.007, indicating a nonlinear relationship between BMI and the improvement of VCSS at 6 weeks after surgery) (Table 3). Through smooth curve fitting, BMI was shown to have a negative relationship trend on the improvement of VCSS at 6 months after surgery (Figure 1).

**Table 3:** Threshold analysis for the relationship between BMI and the improvement of VCSS and AVVQ at six weeks and six months after surgery.

For exposure	Statistics	VCSS improvement (6W)	VCSS improvement (6M)
BMI	(*n* = 170)	β^a^ (95% CI) *p* value	β^a^ (95% CI) *p* value
Model I			
Linear regression coefficient		0.1 (−0.1, 0.2) 0.465	−0.1 (−0.2, 0.1) 0.302
Model II			
Turning point(K)		28	28
<K RC1		−0.1 (−0.3, 0.1) 0.296	−0.2 (−0.3, 0.0) 0.051
>K RC1		0.7 (0.2, 1.2) 0.006*	0.3 (−0.1, 0.7) 0.150
RC2-RC1		0.8 (0.2, 1.4) 0.008*	0.5 (0.0, 1.0) 0.065
Predictive value at turning point		4.1 (3.1, 5.0)	5.1 (4.2, 5.9)
PLLR		0.007*	0.059

*VCSS improvement (6W), the improvement of VCSS at six weeks after surgery; AVVQ improvement (6W), the improvement of AVVQ at six weeks after surgery; VCSS improvement (6M), the improvement of VCSS at six months after surgery; AVVQ improvement (6M), the improvement of AVVQ at six months after surgery; RC, Regression coefficient; PLLR, the* *P* *value of log-likelihood ratio.*

^a^ *Adjusted: Gender; Years; Limbs; Preoperative CEAP.*

*Statistically significant.

*β Beta coefficient.*

After multivariable risk adjustment for potential confounding factors (Table 4), we found that the BMI was not associated with the improvement of VCSS and AVVQ at 6 weeks after surgery, as well as the improvement of AVVQ at 6 months after surgery (all *p*-values >0.05). At 6 months after surgery, we found that the BMI had a negative relationship trend on the improvement of VCSS, and obese patients showed a lower VCSS improvement than patients with normal BMI [−1.3 (−1.9, −0.7) <0.0001].

**Table 4:** Multivariate logistic regression model for risk factors associated with the improvement of AVVQ and VCSS at six weeks and six months after surgery.

Exposure	Non-adjusted	Adjust I	Adjust II
	β^a^ (95% CI) *p* value	β^a^ (95% CI) *p* value	β^a^ (95% CI) *p* value
Y = AVVQ improvement(6W)			
BMI Group			
BMI < 28	0	0	0
BMI ≥ 28	3.1 (−1.6, 7.8) 0.197	−2.8 (−5.6, 0.0) 0.053	−2.9 (−5.6, −0.1) 0.050
Y = AVVQ improvement(6M)			
BMI Group			
BMI < 28	0	0	0
BMI ≥ 28	1.8 (0.6, 1.9) 0.076	0.3 (−1.2, 1.8) 0.686	0.2 (−1.3, 1.8) 0.749
Y = VCSS improvement(6W)			
BMI Group			
BMI < 28	0	0	0
BMI ≥ 28	0.9 (−0.7, 2.4) 0.287	−0.8 (−1.6, 0.1) 0.069	−0.8 (−1.6, 0.1) 0.068
Y = VCSS improvement(6M)			
BMI < 28	0	0	0
BMI ≥ 28	0.2 (−1.3, 1.6) 0.810	−1.3 (−1.9, −0.6) 0.0002*	−1.3 (−1.9, −0.7) <0.0001*

*VCSS improvement (6W), the improvement of VCSS at six weeks after surgery; AVVQ improvement (6W), the improvement of AVVQ at six weeks after surgery; VCSS improvement (6M), the improvement of VCSS at six months after surgery; AVVQ improvement (6M), the improvement of AVVQ at six months after surgery.*

*Statistically significant.

*a: β were derived from multivariate logistic regression analysis; Adjust I adjust for, Gender; Years; Limbs; Preoperative CEAP; Preoperative VCSS; Preoperative AVVQ; Adjust II adjust for, Gender; Years (smooth); Limbs; Preoperative CEAP; Preoperative VCSS; Preoperative AVVQ (smooth).*

### Postoperative Complications

During the observation period of our study, there were no systemic complications, and no patients received reintervention (Table 5). Meanwhile, there was a very low incidence of leg-specific postoperative complications. Paresthesia was the most common complication in the three groups, followed by wound itching (Table 5). Six weeks after surgery, the BMI groups were compared for overall or specific-aspect postoperative complications, and paresthesia was found to be significantly higher in the obese group compared with the normal BMI/overweight group (*p*-value = 0.00032). At 6 months after surgery, although there were no significant differences in the specific-aspect complications between the BMI groups, the overall complications of the legs were significantly higher in the obese group than in the normal BMI group (*p*-value < 0.001) (Table 5).

**Table 5:** Complications after HLS in an ambulatory care at six weeks and six months after surgery.

BMI Group	Normal (BMI < 24)	Overweight (24 ≤ BMI < 28)	Obese (BMI ≥ 28)	*P*
	*N* = 96	*N* = 59	*N* = 15	
	NO. (%)	NO. (%)	NO. (%)	
Six weeks after surgery				
Systemic complications	0	0	0	-
DVT	0	0	0	-
Pulmonary embolism	0	0	0	-
Leg-overall complications	8 (8.33)	7 (11.86)	6 (40)	0.466
Reintervention	0	0	0	-
Hematoma (thigh hematoma >1 cm)	3 (3.125)	1 (1.69)	0	-
Paresthesia	2 (2.08)	3 (5.08)	4 (26.67)	0.0003*
Superficial phlebitis	1 (1.042)	0	1 (6.67)	0.101
Lower limbs swelling	2 (2.08)	2 (3.39)	0	0.716
Wound itching	0	3 (5.08)	2 (13.33)	0.009*
Skin blistering	0	0	0	-
Wound infection	0	1 (1.69)	0	0.388
Six months after surgery				-
Systemic complications	0	0	0	-
DVT	0	0	0	-
Pulmonary embolism	0	0	0	-
Leg-overall complications	2 (2.08)	0	3 (20)	<0.001*
Reintervention	0	0	0	-
Hematoma (thigh hematoma >1 cm)	0	0	0	-
Paresthesia	1	0	1 (6.67)	0.101
Superficial phlebitis	1	0	1 (6.67)	0.101
Lower limbs swelling	1	0	1 (6.67)	0.101
Wound itching	1 (1.042)	0	0	0.683
Skin blistering	0	0	0	-
Wound infection	0	0	0	-

*DVT deep vein thrombosis.*

*Statistically significant.

## Discussion

Previously, numerous studies have examined the relationship between BMI and the incidence of varicose veins (24–27). A few studies have showed the relationship between BMI and the prognosis of varicose veins after ambulatory HLS surgery. In our prospective cohort study, after multivariable risk adjustment for potential confounding factors, we found that the BMI was independent of VCSS improvement and AVVQ improvement at 6 weeks after surgery, as well as the improvement of AVVQ at 6 months after surgery. At 6 months after surgery, we found that the improvement of VCSS in obese patients was significantly lower than that of the normal BMI or overweight groups. We also found that BMI was negatively correlated with the improvement of VCSS in the non-obese group at 6 weeks or 6 months after surgery. While BMI was positively correlated with the improvement of VCSS in obese patients at 6 weeks after surgery and was negatively correlated with the improvement of VCSS in obese patients at 6 months after surgery.

This result seems to be contradictory, but in fact it is reasonable. The BMI was independent of VCSS improvement and AVVQ improvement at 6 weeks after surgery, we believe that an explanation for this anomaly could be that obese patients have an obvious improvement of preoperative discomfort symptoms after surgery that resulting in no significant difference in AVVQ and VCSS scores. In the same way, this can explain why smooth curve fitting showed that BMI of 28 or higher had a positive relationship trend on the improvement of VCSS at 6 weeks after surgery. The VCSS score of obese patients was higher before surgery and the disappearance of postoperative symptoms made the VCSS score was lower, which leading to the improvement of VCSS at 6 weeks after surgery have an obvious improvement, which needs to be further confirmed by larger well-designed studies. Through curve fitting, we observed that the BMI had a negative relationship trend on the improvement of VCSS at 6 weeks after surgery in the non-obese group, although there was no statistical difference in multivariate analysis, which was consistent with the findings by Casana et al. (28). At six months after surgery, a negative correlation between BMI and the improvement of VCSS was confirmed by multivariate analysis and smoothing curves, which was also consistent with currently reports. Currently, the relationship between BMI and the prognosis of varicose veins surgery is controversial. The possible explanations for these inconsistent results were as follows: (a) different cut-off for BMI values in different investigations; (b) different age of individuals enrolled in different studies; (c) racial heterogeneity of BMI; (d) other factors. That needs to be further confirmed by larger well-designed studies.

Despite our efforts to optimize the experimental design, there are still some deficiencies. First, given the prospective design of our study, our sample size was relatively small. Larger well-designed cohort studies are warranted in the future. Second, our study only illustrates the early and midterm outcomes and does not include the long-term outcomes. Last, we did not include ultrasound follow-up data of the patients, which would have shed more light on the postoperative changes of the vein systems.

## Conclusions

The BMI had no significant effect on the early prognosis of HLS performed in an ambulatory center. The mid-term results showed that the BMI had a negative relationship trend on the prognosis of HLS performed in an ambulatory center. Further, patients with high BMI who received ambulatory HLS for varicose veins had a higher risk of postoperative complications compared with patients with low BMI.

## Data Availability

The original contributions presented in the study are included in the article/supplementary material, further inquiries can be directed to the corresponding author’s.
